# The* NLRP3 rs10754558* Polymorphism Is Associated with the Occurrence and Prognosis of Coronary Artery Disease in the Chinese Han Population

**DOI:** 10.1155/2016/3185397

**Published:** 2016-03-24

**Authors:** Dong Zhou, Xinhong Wang, Tao Chen, Wen Wen, Yang Liu, Yue Wu, Zuyi Yuan

**Affiliations:** ^1^Department of Cardiovascular Medicine, First Affiliated Hospital of Medical School, Xi'an Jiaotong University, 277 Yanta West Road, Xi'an, Shaanxi 710061, China; ^2^Department of Cardiovascular Medicine, 3201 Hospital, 783 Tianhan Road, Hanzhong, Shaanxi 723000, China; ^3^Department of Cardiovascular Medicine, Second Affiliated Hospital of Medical School, Xi'an Jiaotong University, 157 West Five Road, Xi'an, Shaanxi 710004, China; ^4^Key Laboratory of Environment and Genes Related to Diseases, Xi'an Jiaotong University, Ministry of Education, Xi'an, Shaanxi 710061, China

## Abstract

The objective of this study is to investigate the potential association of the* NLRP3 rs10754558* and* CARD8 rs2043211* polymorphisms with the occurrence and prognosis of CAD. Gene polymorphisms were analyzed using the ABI PRISM-Snapshot multiplex method in 515 CAD patients and 401 control subjects. The serum level of IL-1*β* was investigated by ELISA assays. The clinical endpoints were evaluated during a median follow-up period of 32 months. The* NLRP3 rs10754558* gene polymorphism was significantly associated with the occurrence of CAD, while the* CARD8 rs2043211* gene polymorphism was not involved. Patients carrying G allele of* NLRP3 rs10754558* had more severe coronary artery stenosis. Multivariable analysis revealed a significant association of the G allele with major adverse cardiac event. The serum IL-1*β* concentrations in patients with GG genotype were significantly increased compared with those in the patients with CC genotype. Our findings for the first time show that the* NLRP3* rs10754558 polymorphism is involved in the occurrence of CAD in the Chinese Han population; and G allele can effectively predict clinical outcome of CAD. The G allele susceptibility to CAD is maybe associated with the increased level of serum IL-1*β*.

## 1. Introduction

Coronary artery disease (CAD), characterized pathologically by atherosclerosis, is the most important cause of death in most countries and acts as a major economic burden on their public health systems [1]. It has been proved that chronic inflammation of the arterial wall is a key element in the pathogenesis of atherosclerosis [2], and the NOD-like receptor pyrin domain containing 3 (NLRP3) inflammasome is conducive to the occurrence of the chronic inflammation [3].

As a multiprotein complex, NLRP3 inflammasome includes the NLRP3 polypeptide, caspase recruitment domain-containing protein 8 (CARD8), the PYD and CARD domain-containing protein (PYCARD), and caspase-1. NLRP3 inflammasome forms and is activated when pattern-recognition receptors detect endogenous and exogenous “danger signals” such as cholesterol crystals [3]. The process results in the NF*κ*B-mediated transcription of pro-IL-1 which leads to accelerating the atherosclerosis progression. NLRP3 inflammasome is significantly upregulated in myocardial fibroblasts and plays a critical role in increasing the infarct size during ischemia-reperfusion [4].

NLRP3 polypeptide and CARD8 control the activity of inflammatory caspase-1 through the formation of NLRP3 inflammasome complexes [5] and are involved in the regulation of caspase-1-mediated IL-1*β* activation [6, 7]. As revealed in a study based on Kawasaki Disease mouse model, caspase-1 and IL-1 are critical inflammatory cytokines in the development of coronary lesions [8]. Genetic variability can affect the function of NLRP3 inflammasome and lead to the change of susceptibility to chronic inflammatory diseases [9]. Numerous studies have demonstrated the association of the gene polymorphisms of* NLRP3 rs10754558* and* CARD8 rs2043211* with susceptibility to diseases such as aspirin-induced asthma [10], abdominal aortic aneurysms [9], and common inflammatory disorder [11]. Meanwhile, a truncating polymorphism (*rs2043211*) can give rise to a nonfunctional CARD8 and induce an amplification of the inflammatory process [12]. It is also proved that* CARD8 rs2043211* polymorphism is significantly associated with susceptibility to gout in Chinese Han males [13]. However, the association of the polymorphisms of* NLRP3 rs10754558* and* CARD8 rs2043211* with CAD has not been reported. Based on the results of previous studies, we hypothesized that the polymorphisms of* NLRP3 rs10754558* and* CARD8 rs2043211 *might be related to the occurrence of CAD and the prognosis of CAD after percutaneous coronary intervention (PCI).

## 2. Materials and Methods

### 2.1. Study Population

This study was approved by the Ethics Committee of the First Affiliated Hospital of Xi'an Jiaotong University (Xi'an, Shaanxi, China) in accordance with the Declaration of Helsinki guidelines. All participants were fully aware of the research and signed informed consents.

In the case-control study, a total number of 916 consecutive Chinese Han subjects (aged from 24 to 80 years) were admitted to the Department of Cardiology at the First Affiliated Hospital of Xi'an Jiaotong University from January 2012 to April 2014 because of chest pain. Based on medical history, typical electrocardiogram changes, myocardial enzymes, and coronary angiography (CAG), the subjects were classified into the CAD group (*n* = 515) and the control group (*n* = 401). The controls were diagnosed to be intercostal neuritis, neurosis or menopause, and regurgitant or oesophagitis.

The prospective cohort study consisted of the 515 CAD patients. All the patients underwent a PCI procedure because of serious coronary artery stenosis and were followed up for a median period of 32 months (range, 14–40 months). Major adverse cardiovascular events (MACEs) are defined as nonfatal MI, cardiovascular death, unstable angina, nonfatal ischemic stroke, and revascularization procedure. According to whether MACEs occurred during the follow-up, the patients were divided into the MACE group and the non-MACE group.

Patients with any of the following were excluded from the study: acute infections, postrevascularization, serious liver or kidney disease, cancer, or acute stroke.

### 2.2. Clinical Measurements

Clinical data of the subjects was collected by well-trained investigators. A history of smoking was defined as either past or current use of cigarettes. Hypertension was defined as systolic blood pressure ≥140 mmHg and/or diastolic blood pressure ≥90 mmHg. Diabetes mellitus (DM) was defined as an elevated fasting plasma glucose concentration >126 mg/dL, or serum glycosylated hemoglobin A1C (HbA1C) level ≥6.5%. The weight (kg) and height (m) of every subject were acquired during the initial visit. Body mass index (BMI) was calculated by the equation: weight (kg)/height (m^2^).

### 2.3. Coronary Angiography and PCI Procedure

Coronary angiographies and PCI procedure were performed according to the Judkins technique. CAD was determined by coronary angiographies with an epicardial coronary artery narrowing of ≥50%. The severity of stenosis in the coronary artery was reflected by Gensini scores [14]. All patients were provided with target vessel revascularization and implanted coronary stent in severe coronary artery with more than 70% luminal narrowing.

### 2.4. Laboratory Analysis

All blood samples (5 mL) were collected into tubes containing anticoagulant and then were centrifuged for 10 minutes at 3000 rpm. Levels of serum high sensitivity C-reactive protein (hs-CRP), total cholesterol (TC), high-density lipoprotein cholesterol (HDL-C), and triglyceride (TG) were measured at the laboratory of our hospital. Low-density lipoprotein cholesterol (LDL-C) was calculated using the Friedewald formula. Interleukin-1*β* (IL-1*β*) in serum was determined by ELISA kits (R&D Systems, Minneapolis, MN, USA).

### 2.5. SNP Selection and Genotyping

In the process of SNP selection of NLRP3 inflammasome, we included in our study those SNPs with a MAF (Minor Allele Frequency) >0.05 in the Chinese Han population. MAFs were analyzed using online tools (http://www.ncbi.nlm.nih.gov/projects/SNP/). Finally, two SNPs (the* NLRP3 rs10754558* and* CARD8 rs2043211*) were selected for further study. Genomic DNA was extracted from white blood cells using the commercially available DNA isolation kit (Tiangen Biotech, Beijing, China) according to the manufacturer's instructions. Genotypes for individual DNA samples were genotyped using the ABI PRISM-Snapshot method (Applied Biosystem, CA, USA). More detail information was shown in Supplementary Materials. Genotypes of 2 SNPs were identified by capillary electrophoresis (ABI PRISM3730 DNA Sequencer; Applied Biosystems). The results were analyzed with GeneMapper 3.0 software (Applied Biosystems). All SNapShot and PCR primers were listed in Supplementary Materials available online at http://dx.doi.org/10.1155/2016/3185397.

### 2.6. Follow-Up

The 515 CAD patients were followed up at months 1, 6, 12, 24, 36, and 40 (median follow-up time 32 months) after discharge and 12 subjects (2.33%) were lost to follow-up. Information of the patients was obtained through telephone communications or face-to-face interviews with the patients or their family members by a trained research cardiologist using a structured questionnaire. MACE incidence of subjects of different genotypes was calculated.

### 2.7. Statistical Analysis

Categorical variable was presented as *n* (%) and continuous variables were presented as mean ± SD. Data from all groups were compared using the *χ* ^2^ test for categorical variables and the independent-samples *t*-test for continuous variables. The odds ratio (OR) and 95% confidence interval (CI) were calculated using logistic regression to ascertain the relative risks of various genotypes for CAD. The association between different genotypes and MACE risk was estimated by Kaplan-Meier survival and Cox regression analyses. The hazard ratio (HR) was presented with 95% CI to show the risk of an event when a genotype was present. All computations were performed with SPSS software 17.0 (SPSS Inc., Chicago, IL). Statistical significance was accepted at the *P* < 0.05 level using a two-tailed test.

## 3. Results

### 3.1. Clinical Characteristics of the Subjects

The clinical characteristics of the 401 control subjects and 515 CAD patients are summarized in Table 1. The prevalence of the risk factors for CAD, such as smoking, DM, and hypertension, was significantly higher in the CAD group than in the control group. The CAD group also had obviously higher levels of BMI, serum Hs-CRP, and LDL-C, but lower levels of HDL-C.

### 3.2. Genotype Frequencies of the* NLRP3 rs10754558* and* CARD8 rs2043211* Polymorphisms

The distributions of the genotypes are shown in Table 2. *χ* ^2^ test confirmed that the genotype frequencies in the control group were concordant with the Hardy-Weinberg equilibrium expectations. The MAFs of* NLRP3 rs10754558* and* CARD8 rs2043211* in our study population were 0.43 and 0.34, respectively. After adjustment of age, gender, hypertension, DM, smoking, LDL-C, and HDL-C, multivariate logistic regression analyses resulted in a significant association between* NLRP3 rs10754558* and CAD (GG versus CC: AOR = 1.630, 95% CI = 1.080–2.459; CC versus CG + GG: AOR = 1.371, 95% CI = 1.024–1.835; CC + CG versus GG: AOR = 1.392, 95% CI = 0.965–2.007; G allele versus C allele: AOR = 1.263, 95% CI = 1.041–1.534). The* CARD8 rs20432111* gene polymorphism was not significantly different between the control subjects and CAD patients (Table 2).

Since the MAF of* rs2043211* is low in Chinese population and the polymorphism of rs2043211 was not associated with CAD, we calculated the statistical power. The statistical power calculation for the case-control analysis was performed using Power and Sample Size Program (http://biostat.mc.vanderbilt.edu/wiki/Main/PowerSampleSize). The study of* rs12976445* has 71% power to detect convincing association with MAF = 0. 34, *α* = 0.05, OR = 1.38.

### 3.3. Relationships between the* NLRP3 rs10754558* and* CARD8 rs2043211* Polymorphisms and the Severity of Coronary Lesions

**Figure 1:**
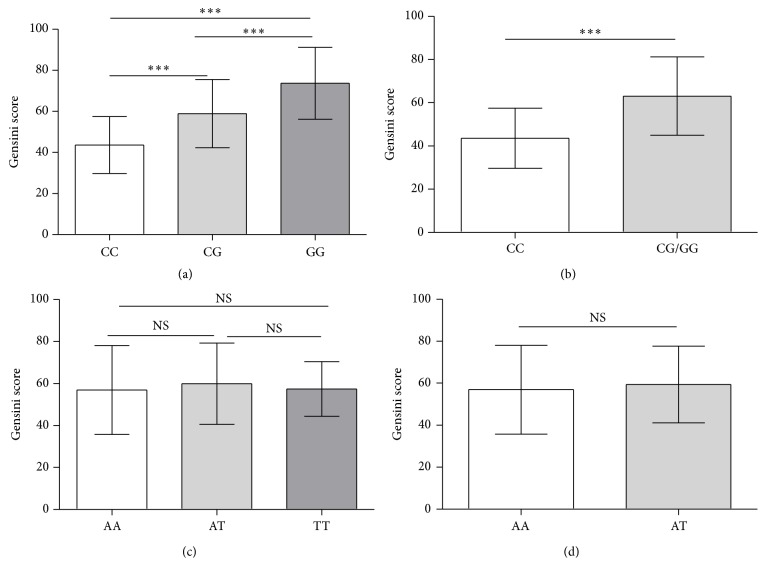
The Gensini scores for* NLRP3 rs10754558* and* CARD8 rs20432111* in CAD patients with different genotypes. Data are shown as mean ± SEM. (a) The Gensini scores for* NLRP3 rs10754558* of 149 patients with CC genotype, 263 with CG genotype, and 103 with GG genotype. (c) The Gensini scores for* CARD8 rs20432111* of 210 patients with AA genotype, 244 with AT genotype, and 61 with TT genotype. ^*∗∗∗*^ *P* < 0.001, ^NS^ *P* > 0.05.

Gensini scoring system is a well-used method for evaluating the severity of coronary atherosclerosis based on angiographic findings. Gensini scores for* NLRP3 rs10754558* and* CARD8 rs20432111* in patients with different genotypes are shown in Figure 1. For* NLRP3 rs10754558*, the mean Gensini scores of CC, CG, and GG genotypes were 43.58 ± 13.86, 58.90 ± 16.65, and 73.67 ± 17.51, respectively. For* CARD8 rs2043211*, the mean Gensini scores of AA, AT, and TT genotypes were 56.93 ± 21.13, 59.89 ± 19.38, and 57.39 ± 12.84, respectively. For* NLRP3 rs10754558*, the score of GG genotype was significantly higher than that of CC and CG genotypes (*P* < 0.001), and the score of CG genotype was also significantly higher than that of CC genotype (*P* < 0.001). This indicated that patients carrying G allele of* NLRP3 rs10754558* had more severe coronary artery stenosis. As to the scores of the genotypes for* CARD8 rs20432111*, no significant difference was identified.

### 3.4. Follow-Up Findings

**Figure 2:**
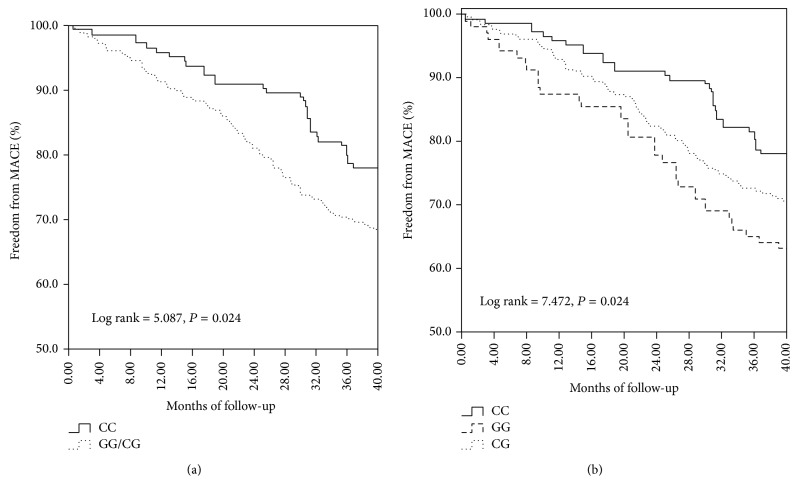
Kaplan-Meier survival curves of the freedom of MACEs and death in CAD patients with different* NLRP3 rs10754558* genotypes. (a) Curves of the freedom of MACEs in dominant model (CC versus CG/GG); (b) curves of the freedom of MACEs in CC, CG, and GG genotypes.

Among 503 patients who accepted follow-up during a median follow-up period of 32 months, 145 (28.83%) had MACEs including 33 deaths, 19 acute myocardial infarctions, 23 coronary revascularizations, 51 unstable anginas, and 19 strokes. The clinical characteristics of patients with or without MACE are summarized in Table 3. As shown in Table 4, Cox regression analysis after the adjustment of age, gender, hypertension, DM, smoking, and hyperlipidemia showed that the* NLRP3 rs10754558* polymorphism was associated with the incidence of MACE in CAD patients and G allele was an independent predictor of such an event (GG versus CC: adjusted HR = 1.790, 95% CI = 1.107–2.893; CC versus CG + GG: adjusted HR = 1.585, 95% CI = 1.065–2.359; G allele versus C allele: adjusted HR = 1.502, 95% CI = 1.182–1.909). However, the distribution of* CARD8 rs20432111* polymorphism was not significantly different between the MACE and non-MACE groups (Table 4). Kaplan-Meier curve demonstrated that patients with GG and/or CG genotype of* NLRP3 rs10754558* tended to have a higher incidence of MACE compared with those with CC genotype (Figures 2(a) and 2(b)). During the follow-up, the MACE incidence in the GG genotype of* NLRP3 rs10754558* was 36.89%, significantly higher than 22.07% in the CC genotype (*P* = 0.015).

### 3.5. The Functional Relevance of the* NLRP3 rs10754558* Polymorphism with the Level of Serum IL-1*β*

**Figure 3:**
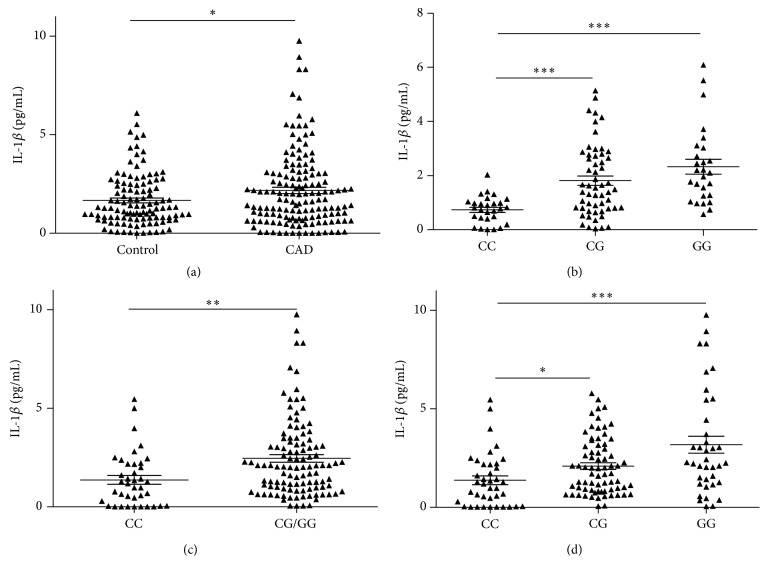
The comparison of the serum levels of IL-1*β* (pg/mL). (a) IL-1*β* concentration in CAD patients and controls. (b) IL-1*β* concentration in controls with different genotypes of NLRP3 rs10754558. (c) IL-1*β* concentration in CAD patients with different genotypes of NLRP3 rs10754558. (d) IL-1*β* concentration in CAD patients with CC and CG/GG genotypes of NLRP3 rs10754558. CG/GG means CG combined with GG genotype. *∗∗∗* indicates *P* < 0.001; *∗∗* indicates *P* < 0.01; *∗* indicates *P* < 0.05 (versus CC genotype).

In order to identify the pathogenic mechanisms of the* NLRP3 rs10754558* polymorphism, ELISA analysis was performed to detect the level of serum IL-1*β* in 148 patients as well as 111 healthy individuals. As shown in Figure 3(a), the IL-1*β* concentrations in the serum of patients with CAD were significantly increased compared to control subjects (mean ± SD = 2.177 ± 1.921 versus 1.668 ± 1.319 pg/mL) (*P* = 0.017). Compared with CC genotype (*n* = 28) in control subjects, the serum content of IL-1*β* was significantly increased in control subjects with GG genotypes (*n* = 27) (Figure 3(b), mean ± SD = 2.328 ± 1.423 versus 0.739 ± 0.493 pg/mL, *P* < 0.001). As shown in Figure 3(c), the IL-1*β* concentrations of CAD patients carrying G allele (CG + GG genotype, *n* = 109) were obviously higher than those in patients with CC genotype (*n* = 39) (mean ± SD = 2.467 ± 2.011 versus 1.367 ± 1.367 pg/mL, *P* = 0.002). Moreover, the serum IL-1*β* concentrations were significantly increased in patients with GG genotype (*n* = 37) (mean ± SD = 3.204 ± 2.684 versus 1.367 ± 1.367 pg/mL, *P* < 0.001) compared with the patients with CC genotype (*n* = 39) (Figure 3(d)). These results implied that the IL-1*β* concentrations of subjects carrying G allele of NLRP3 rs10754558 had an obvious increase compared with subjects carrying C allele.

## 4. Discussion

In this study, our current results demonstrate a clear correlation between the* NLRP3 rs10754558* gene polymorphism and CAD. Individuals carrying the G allele of* rs10754558* have an obviously higher risk of developing CAD than those without a G allele (OR ranging from 1.041 to 1.534). The G allele also increases the risk of MACE in CAD patients (HR = 1.502). The G allele of* NLRP3 rs10754558* polymorphism was associated with increased IL-1*β* levels in the sera of subjects. Meanwhile, our results show no significant association of the* CARD8 rs10754558* gene polymorphism with CAD and its prognosis.

Atherosclerosis is the most important pathophysiological characteristic of CAD. There is a consensus that inflammatory response plays an important role in the progression of atherosclerosis. Many kinds of inflammatory cytokines, such as IL-1*β*, are involved in inflammatory response of atherosclerosis. Inflammasome can facilitate the production and maturation of several important proinflammatory cytokines and their involvement in the chronic inflammation that underlies atherogenesis in vessel walls [5, 15]. Activation of NLRP3 inflammasome is an important step in the progression of atherosclerosis-related inflammation [15, 16]. NLRP3 and CARD8 control the assembly of NLRP3 inflammasome which is a crucial molecular platform regulating activation of caspase-1 and processing of IL-1*β* [5]. Clinical studies have shown that the* mRNA* level of NLRP3 in PBMCs from CAD patients is positively correlated with the plasma level of IL-1*β* [17, 18]. Knockdown of NLRP3 can alleviate the production of IL-1*β* [17].

Research has shown that the gene polymorphism of NLRP3 rs10754558 is associated with common inflammatory diseases or a known functional effect [10, 11]. The G allele of* rs10754558* polymorphism changed NLRP3 mRNA stability [10]. Moreover, the allele-specific construct containing the G allele of the* rs10754558* shows a 1.3-fold higher activity than that containing the C allele [10]. The polymorphism of NLRP3 influences the inflammasome activation in atherosclerosis and in turn the susceptibility of people to CAD. The data in study reveals for the first time that patients with the GG genotype of* NLRP3 rs10754558* are more prone to CAD and suffer poor prognosis after PCI. Meanwhile, the IL-1*β* concentrations of subjects carrying G allele of* NLRP3 rs10754558* had an obvious increase compared with subjects carrying C allele. These results suggest that the G allele of NLRP3 rs10754558 may be involved in the progression of atherosclerosis in CAD patients and the pathogenicity of the G allele of the* NLRP3 rs10754558* was maybe associated with the level of IL-1*β* in serum.

CARD8 has been proved to be associated with several inflammatory diseases [9, 19]. A previous study reported a significantly increased CARD8 mRNA expression in human atherosclerotic plaques compared with the control tissues from transplantation donors [20]. However, knockdown of CARD8 did not affect the expression of IL-1*β* and IL-1Ra mRNA or the release of IL-1*β* protein [21]. A recent study suggests that there is no significant association between the gene polymorphism of* CARD8 rs20432111* and the mRNA expression of IL-1*β*, TNF, or IL-18 [20]. In fact, previous studies on the relationship between the gene polymorphism of* CARD8 rs20432111* and chronic inflammatory diseases have generated conflicting results. The presence of the T allele of* CARD8 rs20432111* could play a potential protective role in Crohn's disease [22]. However, the T allele is also reported to be related to an increased risk of rheumatoid arthritis and inflammatory bowel diseases [23, 24]. Our study identifies no association between the gene polymorphism of* CARD8 rs20432111* and CAD, which is consistent with the results of some previous studies [20, 25].

Even though we carried out a comprehensive analysis, some limitations of this study need to be noted in evaluating our results. The sample size was relatively small. Further studies with larger sample sizes, longer follow-up periods, and more detailed examination of the mechanism of G allele in atherosclerosis should be conducted to assess the effect of* NLRP3 rs10754558* gene polymorphism on CAD.

In conclusion, the present study shows that the G allele of* NLRP3 rs10754558* is associated with the occurrence and poor prognosis of CAD in Chinese Han population. The G allele susceptibility to CAD is maybe associated with the increased level of serum IL-1*β*.

## Supplementary Material

DNA Isolation and Genotyping.

## Figures and Tables

**Table 1:** Baseline characteristics of the control and CAD subjects.

	Control	CAD	*P*
Total, *n*	401	515	
Male, *n* (%)	241 (60.10%)	364 (70.68%)	0.001
Age, y (mean ± SD)	57.49 ± 10.67	59.50 ± 11.53	0.031
Hypertension (%)	194 (48.38%)	286 (55.53%)	0.033
DM (%)	69 (17.21%)	132 (25.63%)	0.002
Smoking (%)	151 (37.66%)	248 (48.16%)	0.002
SBp (mmHg)	127.16 ± 19.22	125.53 ± 22.99	0.242
DBp (mmHg)	79.26 ± 12.73	78.48 ± 14.25	0.389
HR (beats/min)	72.75 ± 13.72	72.86 ± 16.98	0.912
BMI (kg/m^2^)	24.18 ± 2.86	24.75 ± 2.86	0.003
LVEF (%)	63.06 ± 11.85	57.65 ± 12.12	0.156
Hs-CRP (mg/L)	2.71 ± 0.98	6.38 ± 2.51	<0.001
TC (mmol/L)	3.83 ± 0.85	3.95 ± 0.96	0.051
TG (mmol/L)	1.65 ± 0.82	1.70 ± 0.74	0.334
HDL-C (mmol/L)	1.00 ± 0.25	0.96 ± 0.25	0.016
LDL-C (mmol/L)	2.16 ± 0.77	2.31 ± 0.79	0.003

CAD: coronary artery disease; SBp: systolic blood pressure; DBp: diastolic blood pressure; HR: heart rate; BMI: body mass index; LVEF: left ventricular ejection fraction; Hs-CRP: high-sensitivity C-reactive protein; TC: total cholesterol; TG: triglyceride; HDL-C: high-density lipoprotein cholesterol; LDL-C: low-density lipoprotein cholesterol; DM: diabetes mellitus.

**Table 2:** Genotype frequencies of *NLRP3 rs10754558* and* CARD8 rs2043211* in the control and CAD subjects.

	Control	CAD	AOR (95% CI)^*∗*^	*P*
	(*n* = 401)	(*n* = 515)		
*NLRP3 rs10754558*				
CC	142 (35.41%)	149 (28.93%)	1.000 (reference)	
CG	200 (49.88%)	263 (51.07%)	1.294 (0.952–1.758)	0.099
GG	59 (14.71%)	103 (20.00)	1.630 (1.080–2.459)	0.020
Dominant model (CC versus CG + GG)			1.371 (1.024–1.835)	0.034
Recessive model (CC + CG versus GG)			1.392 (0.965–2.007)	0.077
C allele	484 (60.35%)	561 (54.47%)	1.000 (reference)	
G allele	318 (39.65%)	469 (45.53%)	1.263 (1.041–1.534)	0.018
HWE χ^2^	0.713	0.450		
HWE *P*	0.399	0.502		

*CARD8 rs2043211*				
AA	175 (43.64%)	210 (40.78%)	1.000 (reference)	
AT	186 (46.38%)	244 (47.38%)	1.160 (0.870–1.545)	0.311
TT	40 (9.98%)	61 (11.84%)	1.252 (0.787–1.990)	0.343
Dominant model (AA versus AT + TT)			1.176 (0.894–1.547)	0.245
Recessive model (AA + AT versus TT)			1.158 (0.747–1.795)	0.513
A allele	536 (66.83%)	664 (64.47%)	1.000 (reference)	
T allele	266 (33.17%)	366 (35.53%)	1.126 (0.921–1.377)	0.248
HWE χ^2^	0.858	0.600		
HWE *P*	0.354	0.439		

CAD: coronary artery disease; AOR: adjusted odds ratio; HWE: Hardy-Weinberg equilibrium; 95% CI: 95% confidence interval.

^*∗*^The AOR on the basis of risk factors such as age, sex, hypertension, diabetes mellitus, hyperlipidemia, and smoking.

**Table 3:** Baseline characteristics of CAD patients with and without MACE during follow-up.

	Patients without MACE	Patients with MACE	*P*
Total, *n*	358	145	
Male, *n* (%)	240 (67.04%)	118 (81.38%)	0.001
Age, y (mean ± SD)	58.52 ± 11.58	61.75 ± 11.05	0.004
Hypertension (%)	188 (52.51%)	93 (64.14%)	0.018
DM (%)	77 (21.51%)	52 (35.86%)	0.001
Smoking (%)	159 (44.41%)	84 (57.93%)	0.008
SBp (mmHg)	125.79 ± 23.71	125.34 ± 21.64	0.843
DBp (mmHg)	78.73 ± 14.16	78.39 ± 14.01	0.802
HR (beats/min)	73.44 ± 16.92	71.37 ± 16.79	0.212
BMI (kg/m^2^)	24.63 ± 2.71	25.20 ± 3.22	0.045
LVEF (%)	57.58 ± 12.03	55.54 ± 11.79	0.085
Hs-CRP (mg/L)	5.76 ± 2.24	6.34 ± 3.64	0.029
TC (mmol/L)	3.91 ± 0.95	4.09 ± 0.99	0.060
TG (mmol/L)	1.69 ± 0.72	1.73 ± 0.82	0.590
HDL-C (mmol/L)	0.99 ± 0.25	0.88 ± 0.22	<0.001
LDL-C (mmol/L)	2.26 ± 0.74	2.51 ± 0.87	0.001
ACE-I/ARB, *n* (%)	247 (68.99%)	96 (66.21%)	0.527
Statins, *n* (%)	343 (95.81%)	134 (92.41%)	0.124
*β*-blocker, *n* (%)	246 (68.72%)	105 (72.41%)	0.454
Aspirin, *n* (%)	344 (96.09%)	137 (94.48%)	0.471

CAD: coronary artery disease; MACE: major adverse cardiacevent; SBp: systolic blood pressure; DBp: diastolic blood pressure; HR: heart rate; BMI: body mass index; LVEF: left ventricular ejection fraction; Hs-CRP: high-sensitivity C-reactive protein; TC: total cholesterol; TG: triglyceride; HDL-C: high-density lipoprotein cholesterol; LDL-C: low-density lipoprotein cholesterol; DM: diabetes mellitus; ACEI: angiotensin converting enzyme inhibitor; ARB: angiotensin receptor antagonist.

**Table 4:** Genotype frequencies of *NLRP3 rs10754558* and *CARD8 rs2043211* gene polymorphisms in CAD patients with and without MACE during follow-up.

	Patients without MACE	Patients with MACE	Adjusted HR (95% CI)^*∗*^	Log-rank *P*
	(*n* = 358)	(*n* = 145)		
*NLRP3 rs10754558*				
CC	113 (31.56%)	32 (22.07%)	1.000 (reference)	
CG	180 (50.28%)	75 (51.72%)	1.503 (0.990–2.282)	0.056
GG	65 (18.16%)	38 (26.21%)	1.790 (1.107–2.893)	0.017
Dominant model (CC versus CG + GG)			1.585 (1.065–2.359)	0.023
Recessive model (CC + CG versus GG)			1.363 (0.934–1.989)	0.108
C allele	406 (56.70%)	139 (47.93%)	1.000 (reference)	
G allele	310 (43.30%)	151 (52.07%)	1.502 (1.182–1.909)	0.001
HWE χ^2^	0.206	0.191		
HWE *P*	0.650	0.662		

*CARD8 rs2043211*				
AA	146 (40.78%)	60 (41.38%)	1.000 (reference)	
AT	168 (46.93%)	68 (46.90%)	1.193 (0.831–1.711)	0.338
TT	44 (12.29%)	17 (11.72%)	0.850 (0.483–1.497)	0.574
Dominant model (AA versus AT + TT)			1.111 (0.786–1.571)	0.551
Recessive model (AA + AT versus TT)			1.044 (0.981–1.111)	0.174
A allele	460 (64.25%)	188 (64.83%)	1.000 (reference)	
T allele	256 (35.75%)	102 (35.17%)	0.992 (0.774–1.272)	0.949
HWE χ^2^	0.165	0.117		
HWE *P*	0.685	0.733		

CAD: coronary artery disease; HR: hazard risk; MACE: major adverse cardiovascular event. HWE: Hardy-Weinberg equilibrium; 95% CI: 95% confidence interval.

^*∗*^The adjusted HR on the basis of risk factors such as age, sex, hypertension, diabetes mellitus, hyperlipidemia, and smoking.
