# Supplementary material for: The NLRP3 rs10754558 Polymorphism Is Associated with the Occurrence and Prognosis of Coronary Artery Disease in the Chinese Han Population
Source: Biomed Res Int. 2016 Mar 24;2016:3185397. doi: 10.1155/2016/3185397 (PMC4823501; doi:10.1155/2016/3185397)
Supplement: Supplementary file 1 — DNA Isolation and Genotyping. [file 3185397.f1.pdf]

DNA Isolation and Genotyping

White blood cells and serum were separated and stored at -80℃.Genomic DNA was extracted from the white blood cells using the commercially available DNA isolation kit (Tiangen Biotech, Beijing, China) according to the manufacturer's protocol. Genotyping was performed for each individual DNA sample using the ABI PRISM-Snapshot method (Applied Biosystem, USA). In brief, the Snapshot reactions were performed in a 10 μl final volume containing primer mix (0.02-0.5μmol/l), 5μl Snapshot Multiplex Ready Mix, and 4μl template consisting of the multiplex PCR products. The multiplex PCR products were purified with the Fermentas PCR Purification Kit (Fermentas, CA) following a cycling program consisted of 25 cycles of 96℃ for 10s, 51℃ for 5s, and 60℃ for 30 s. The extension products were purified by a 15-min incubation with shrimp alkaline phosphatase (1 U, Fermentas, CA) at 37℃ and a subsequent 15-min incubation at 75℃ to denature the enzyme. After mixture of the purified products (0.5μl), Hidi-Formamide (8.5μl) and GeneScanTM-120 LIZ Size Standard (Applied Biosystems, 0.5μl), the final reaction mix was denatured at 95℃for 5 min. The genotypes of the *NLRP3 rs10754558* and *CARD8 rs204321* were identified by capillary electrophoresis (ABIPRISM3730 DNA Sequencer; Applied Biosystems). The results were analyzed with Gene Mapper 3.0 software (Applied Biosystems). All the SNapShot and PCR primers are listed in Supplementary Table 1. Finally, to ascertain the multiplex Snapshot results, 40 samples were selected at random and re-genotyped by direct sequencing using a BigDye terminator (Applied Biosystem). All assays were 100% concordant.

Supplemenary Table 1. Snapshot and PCR primers of the gene polymorphisms of the NLRP3 rs10754558 and CARD8 rs2043211

Gene	polymorphism	Forward primer	Reverse primer	SNaPshot primer
CARD8	rs20432111	ATTGAGACACAGCGTCCAATAG	ATGCTATCATCAGGCACCTACC	ttttttttttttttttttttttttACTCAGGAACAGCACGGA
NLRP3	rs10754558	CCTGAGCTGACCGTCGTCTT	ATGAGGTCACCAAGAGGAACATC	ttttttttttttttttttttttttttttttACAGCATCGGGTGTTGTT
